# Genome Sequencing of a Mung Bean Plant Growth Promoting Strain of *P. aeruginosa* with Biocontrol Ability

**DOI:** 10.1155/2014/123058

**Published:** 2014-08-12

**Authors:** Devaraj Illakkiam, Manoharan Shankar, Paramasivan Ponraj, Jeyaprakash Rajendhran, Paramasamy Gunasekaran

**Affiliations:** ^1^Department of Genetics, School of Biological Sciences, Madurai Kamaraj University, Madurai, Tamilnadu 625021, India; ^2^Department of Microbiology, Molecular Genetics and Immunology, University of Kansas Medical Center, Kansas City, KS 66160, USA; ^3^Department of Cell and Developmental Biology, John Innes Center, Norwich Research Park, Norwich NR4 7UH, UK

## Abstract

*Pseudomonas aeruginosa* PGPR2 is a mung bean rhizosphere strain that produces secondary metabolites and hydrolytic enzymes contributing to excellent antifungal activity against *Macrophomina phaseolina*, one of the prevalent fungal pathogens of mung bean. Genome sequencing was performed using the Ion Torrent Personal Genome Machine generating 1,354,732 reads (6,772,433 sequenced bases) achieving ~25-fold coverage of the genome. Reference genome assembly using MIRA 3.4.0 yielded 198 contigs. The draft genome of PGPR2 encoded 6803 open reading frames, of which 5314 were genes with predicted functions, 1489 were genes of known functions, and 80 were RNA-coding genes. Strain specific and core genes of *P. aeruginosa* PGPR2 that are relevant to rhizospheric habitat were identified by pangenome analysis. Genes involved in plant growth promoting function such as synthesis of ACC deaminase, indole-3-acetic acid, trehalose, mineral scavenging siderophores, hydrogen cyanide, chitinases, acyl homoserine lactones, acetoin, 2,3-butanediol, and phytases were identified. In addition, niche-specific genes such as phosphate solubilising 3-phytase, adhesins, pathway-specific transcriptional regulators, a diguanylate cyclase involved in cellulose synthesis, a receptor for ferrienterochelin, a DEAD/DEAH-box helicase involved in stress tolerance, chemotaxis/motility determinants, an HtpX protease, and enzymes involved in the production of a chromanone derivative with potent antifungal activity were identified.

## 1. Introduction

*Pseudomonas aeruginosa *is one of the prevalent organisms present in diverse natural environments. It is an aquatic and soil bacterium that can infect a range of hosts including humans in an opportunistic manner [1]. Though they are specially noted for their pathogenicity, the recent research and genomic studies have thrown light into the unique property of these organisms to produce a wide array of secondary metabolites with multiple potential biotechnological applications.* Pseudomonas aeruginosa* has a large number of paralogous groups (distinct gene families), indicating that its genome has evolved through genetic expansion [2]. It has developed adaptation and competitive fitness in a diverse range of ecological niches.* P. aeruginosa* is known to produce bioactive compounds showing antagonistic activity against plant pathogens.* P. aeruginosa* strains such as 7NSK2 [3], PNA1 [4], NJ-15 [5], and PUPa3 [6] have been reported to have plant growth promoting ability and biocontrol activity against phytopathogens.* Pseudomonas aeruginosa* PGPR2 was isolated from the rhizosphere of mung bean plant with the ability to promote plant growth. This strain showed efficient antagonistic activity against* Macrophomina phaseolina*, the causative agent of root rot diseases [7].

Though genome sequences of several strains of medically relevant* P. aeruginosa* have been reported, the genome of only one agriculturally relevant strain,* P. aeruginosa *M18, has been reported until now. Thus, in order to define the differences in the genome structures between* P. aeruginosa* strains from nosocomial and rhizosphere niches, the whole genome of the strain PGPR2 was sequenced and compared with those of previously sequenced medically relevant strains. In this communication we report the genomic regions that are evolutionarily preserved and varied between* P. aeruginosa* PGPR2 and other medically relevant strains. We have comprehensively compared the PGPR2 draft genome with the genomes of six other strains (M18, DK2, LESB58, PA7, PAO1, and UCBPP-PA14). We report the core and niche-specific genome organization in this ubiquitous species.

## 2. Materials and Methods

### 2.1. Bacterial Growth and DNA Extraction

A single colony of* P. aeruginosa *PGPR2 grown on Luria-Bertani (LB) agar was inoculated into 5 mL of LB broth and grown overnight with agitation at 30°C. Bacterial cells were collected by centrifugation and the genomic DNA was isolated using the Qiagen DNeasy kit following the manufacturer's protocol for Gram negative bacteria (Qiagen, Hilden, Germany). The quantity, quality, and integrity of the isolated DNA were verified by spectrophotometry (NanoDrop products, Wilmington, DE, USA) and gel electrophoresis.

### 2.2. Whole Genome Sequencing and Assembly

Genomic libraries were generated using 1 *μ*g of genomic DNA using the Ion Xpress Plus fragment library kit (Life technologies, NY, USA). Briefly, genomic DNA was enzymatically sheared and the quality and quantity were analyzed on a bioanalyzer (Multina, Shimadzu, Japan). Adapters were ligated to the sheared DNA fragments and the fragments were size-selected on 2% E-gel (Lifetechnologies). The size selected library (150 to 300 bp) was used for template preparation on an Ion one-touch automated template preparation system using the Ion One-touch Template 200 kit. Template positive ion sphere particles (ISPs) were enriched using the Ion one touch ES system, loaded onto an Ion 316 chip, and sequenced using the Ion sequencing 200 kit on Ion Torrent Personnel Genome machine. The MIRA assembler v 3.4.1 was used to generate a reference genome assembly of* P. aeruginosa* PGPR2 using* P. aeruginosa* DK2 genome (NC_018080) as template.

The template sequence was removed after alignment and the unaligned reads were extracted for* de novo* assembly using MIRA v 3.4.1 [8]. The resulting contigs of high quality and appreciable length were added to the assembly. The final draft genome was viewed and edited, when required, using Staden Package version 2.0 [9].

### 2.3. Genome Annotation and Comparative Genome Analysis

The PGPR2 genome was annotated using the Rapid Annotation using Subsystems Technology (RAST) server [10]. The annotated genome was compared with other related and distant genomes maintained in the SEED Viewer environment. Blastp was used to find homologs of selected PGPR2 sequences in all the annotated proteins of the* P. aeruginosa* strains, M18, DK2, LESB58, PAO1, UCBPP-PA14, and PA7. Ribosomal RNA and transfer RNA genes were predicted by RNAmmer v1.2 [11] and tRNAScan-SE [12], respectively. A complete set of open reading frames predicted to encode proteins was identified using GLIMMER [13]. Genomes/contigs were aligned with each other using Mummer v 3.20 [14] and Mauve v 2.3.1 [15]. InterProScan was used to identify conserved domains in selected sequences [16]. A comprehensive genome comparison was performed across seven* P. aeruginosa* strains using Gview server and the results were visualized using the WebAct Tool [17]. DNAplotter [18] was used to construct a* P. aeruginosa* PGPR2 genome atlas while CRISPR repeats were identified using the CRISPR finder [19]. The CVtree tool [20] was used to perform phylogenetic analysis of PGPR2 in comparison with other* P. aeruginosa* genomes (M18, PAO1, DK2, LESB58, PA7, and UCBPP-PA14). Strain specific regions on the* P. aeruginosa* PGPR2 genome were detected using Panseq server with default parameter [21]. The metabolic pathways of the strain PGPR2 were constructed and compared with those of other strains using KAAS (KEGG Automatic Annotation Server) [22] and the MetaCyc database [23].

## 3. Results and Discussion

### 3.1. Genome Features

**Figure 1:**
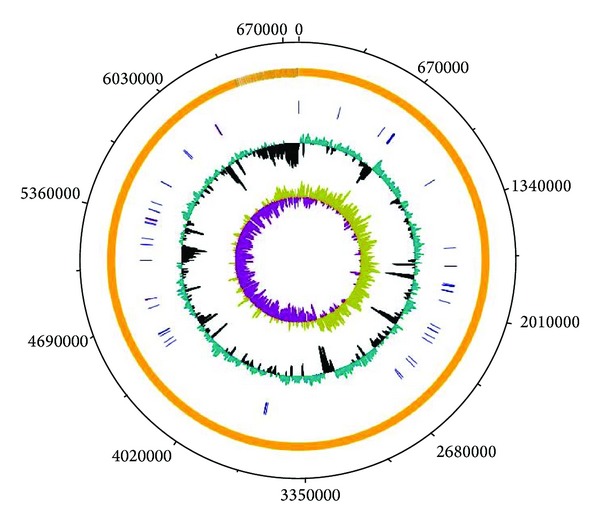
Graphical map of the* P. aeruginosa* PGPR2 draft genome. From the outside to the inside: open reading frames, rRNA operons, and tRNAs are shown in yellow, red, and blue, respectively. G+C content plot and GC skew (purple: negative values, olive: positive values) are also shown.

The mung bean rhizosphere isolate PGPR2 showed efficient plant growth promoting activity and antagonistic activity against* Macrophomina phaseolina*, a common fungal plant pathogen. In this study, we report the whole genome sequence of strain PGPR2 and the genetic factors responsible for plant growth promoting properties. The draft genome of* P. aeruginosa* PGPR2 was 6772433 bp long comprising 198 contigs (Genbank accession number: ASQO00000000). The* P. aeruginosa* PGPR2 genome contains 6803 predicted open reading frames (ORFs), of which 80 were RNA encoding genes, 5314 were protein encoding genes (PEGs) with predicted functions, and 1489 were PEGs with unknown functions. The average GC content of the PGPR2 genome was 66%, which is consistent with previously reported* P. aeruginosa* genomes. Approximately, 86.9% of the total PGPR2 genome was found to be coding regions. The* P. aeruginosa* PGPR2 genome is graphically represented in Figure 1 while the genomic features are summarized in Table 1.

### 3.2. Unique Genomic Regions of* P. aeruginosa* PGPR2 Relevant to Its Rhizospheric Habitat

**Figure 2:**
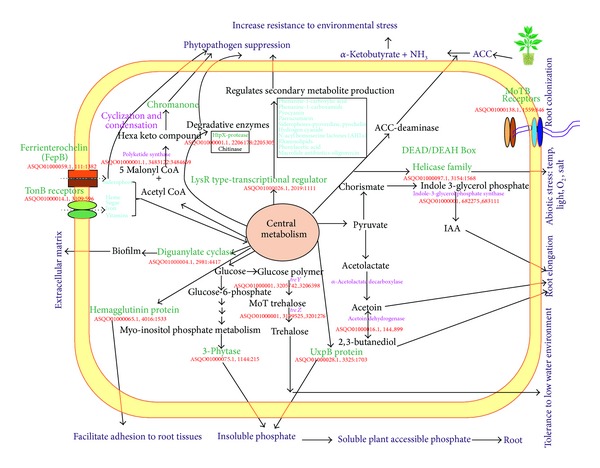
Diagrammatic representations of various unique and core genomic regions of PGPR2 strain relevant to its rhizospheric habitat. Predicted and annotated gene sequences were analyzed for similarity with the NCBI database followed by assignment of each gene into KEGG pathway. Based on individual analysis results of the KEGG pathway, proposed biochemical pathways were constructed which demonstrated characteristic features related to plant growth promotion. Unique genes are indicated by green color.

Plant growth-promoting rhizobacteria have the ability to scavenge mineral nutrients, fix atmospheric nitrogen, solubilize soil phosphorus, and synthesize regulators of plant metabolism [24]. The strain PGPR2 was shown to exhibit effective plant growth promoting properties towards the mung bean plant in both* in vivo* and* in vitro *conditions. This strain also had a strong suppressive effect on the growth of* Macrophomina phaseolina*. Earlier, we have reported a secondary metabolite (3, 4-dihydroxy-N-methyl-4-(4-oxochroman-2-yl) butanamide) and a secreted protease responsible for the antifungal activity [7, 25]. Among several reported strains,* P. aeruginosa *M18 strain was the only reported genome with plant growth promotion and biocontrol activity [26]. Analysis of the genome sequence of PGPR2 revealed several features that reflect this bacterium highlighting its plant growth promoting ability (Figure 2), a few of which are discussed in detail.

### 3.3. 3-Phytase

A 930 bp ORF (Genbank accession number ASQO01000075.1, region 1144 : 215) coding for 3-phytase was identified in PGPR2 with no homologs in other* P. aeruginosa* strains. Sequence analysis of this ORF revealed maximum similarity to 3-phytase from* Pseudomonas protegens *CHAO with 68% identity using BlastP analysis. Rhizospheric bacteria can benefit plant growth by solubilization of inorganic phosphates. This is achieved by conversion of insoluble phosphorus (P) to an accessible form* via *several enzymes. Nonspecific phosphatases are known to perform dephosphorylation of phosphoester or phosphoanhydride bonds in organic matter [27]. Phytases on the other hand specifically release P from phytic acid [28]. Phosphonatases and C-P Lyases perform C-P cleavage in organophosphonates [29]. All other analyzed* P. aeruginosa* strains do not produce this enzyme including* P. aeruginosa *M18, a reported PGPR strain.

### 3.4. TonB-Dependent Receptor and Hemagglutinin

TonB-dependent receptors (TBDRs) allow Gram-negative bacteria to uptake scarce resources from competitive environments with very high affinity. Earlier reports on TBDRs focused on the uptake of siderophore-iron complexes but recent studies have showed that the spectrum of ligands that can be scavenged includes sugars, vitamins, heme, and other nonferrous cations [30]. One of the TBDRs identified in PGPR2 was found to be unique to this strain. A total of 24 TBDRs were identified from the genome of* P. aeruginosa* PGPR2. BlastP analysis revealed a strain specific region (2514 bp; Genbank accession number ASQO01000014.1, regions 3109 : 596) contains a unique TBDR, which showed 98% identity with a TBDR of* Pseudomonas nitroreducens*. Filamentous hemagglutinins are involved in plant attachment and root adhesion of plant associated bacteria thereby indirectly responsible for plant growth promotion [31]. Bacterial attachment to the root surface by various adhesins is an important trait required for competitive colonization. The genome of* P. aeruginosa* PGPR2 encoded adhesin/hemagglutinin protein (2484 bp; Genbank accession number ASQO01000065.1, region 4016 : 1533), possibly involved in the initial adhesion to mung bean roots. This region was found to share 73% identity to the adhesin/hemagglutinin gene of* Pseudomonas protegens* CHA0.

### 3.5. LysR Family Transcriptional Regulator PA2877

Apart from the global regulatory systems, the pathway-specific regulators are responsible for the transcriptional activation of the secondary metabolite biosynthetic operons. LysR family response regulators have been shown to be involved in the positive regulation of antifungal metabolite production [32]. PGPR2 strain encoded a LysR family transcriptional regulator (909 bp; Genbank accession number ASQO01000026.1, regions 2019 : 1111), which showed maximum homology with a LysR family regulator from* Achromobacter xylosoxidans *(68% identity) in BlastP analysis. Pfam domain analysis identified a 60-amino acid helix-turn-helix domain (PF00126.22), which is known to be involved in DNA binding and 209-amino acid as LysR substrate binding domain (PF03466.15).

### 3.6. Diguanylate Cyclase

Diguanylate cyclases usually possess nucleotide cyclase activity and aid in cyclic-di-GMP formation. Cyclic-di-GMP signaling is used by many bacteria to regulate vital functions such as biofilm formation [33]. Since biofilm formation is an important survival strategy for many bacteria, the synthesis and degradation of cyclic-di-GMP is tightly regulated by diguanylate cyclases, which contain domains with conserved GGDEF and EAL sequence motifs. Most sequenced bacterial genomes contain several GGDEF motif containing proteins. The GGDEF domain is usually responsible for diguanylate cyclase activity and also for cellulose synthase activity. Cellulose fibres in well-documented PGPR (*Agrobacterium *sp. and* Rhizobium* sp.) help in anchoring of bacteria on plant surfaces, thus facilitating permanent colonization and establishment of biofilm-like structures on the root surface [34]. PGPR2 contained a unique region (1437 bp; Genbank accession number ASQO01000004.1, regions 2981 : 4417) encoding a diguanylate cyclase with the characteristic “GGDEF” domain. This gene shared 78% identity with a homolog from* Pseudomonas putida* using BlastP analysis.

### 3.7. UxpB Protein

The genome of PGPR2 harbours a strain specific region (1623 bp; Genbank accession number ASQO01000028.1, regions 3325 : 1703) coding for a UxpB homolog. This region shared 88% identity with the* Pseudomonas denitrificans* ATCC 13867 genome using BlantN analysis and TblastN showed homology with the same organism but with 51% identity. Earlier reports have demonstrated that UxpB and its homologs are induced under inorganic phosphate (P_i_) limitation and thus enable the utilization of various organic phosphate sources. Expression of this gene helps the plants in accessing the scarce nutrients that are normally difficult to obtain, while limiting their concentration in the niche for competing pathogens [35].

### 3.8. Alpha/Beta-Hydrolase Fold Family Enzyme

A strain specific region (Genbank accession number ASQO01000004.1, regions 1492 : 2373) encoding a 293-amino acid protein, homologous to a hydrolytic enzyme containing an alpha/beta-hydrolase fold from* Pseudomonas* sp. GM25 with 92% identity in BlastP analysis was identified. Earlier studies have shown that members of the alpha/beta-hydrolase fold family are capable of cleaving the lactone ring of acylhomoserine lactones [36].* N*-Acylhomoserine lactones (AHLs) are signaling molecules in quorum-sensing (QS) systems that regulate virulence, disease incidence, and establishment in pathogenic bacteria. The ability to cleave these signals (quorum quenching) and disrupt communication in the rhizosphere will help PGPR2 to nullify the advantage of population in competing pathogens.

### 3.9. Chemotaxis/Motility

Two unique regions (712 bp and 723 bp; Genbank accession number ASQO01000138.1, regions 1559 : 846, 2311 : 1589) from PGPR2 strain were identified that showed homology to corresponding homologs from* Pseudomonas *sp. UW4 in BlastP analysis with 95 and 85% identity, respectively. They possessed highly conserved domains and were annotated as MotB-related protein essential for chemotaxis and methyl-accepting chemotaxis protein (MCPs). Nearly, all motile bacteria have genes encoding the signal transduction pathway for chemotaxis. Earlier studies reported that motility and chemotaxis are important traits for root colonization [37] and a requisite for improved biocontrol activity against the pathogenic fungi. Methyl-accepting chemotaxis proteins (MCPs) are responsible for the detection of ligands, the binding of which initiates a chemotaxis signalling cascade thereby leading to root directed motility.

### 3.10. DEAD/DEAH-Box Helicase

DEAD-box helicases play a key role in tolerance to various abiotic stresses including temperature, light, oxygen, and salt stress. Earlier studies suggested that genes belonging to the DEAD-box helicase family are induced by salinity and function as a typical helicase to unwind DNA and RNA [38]. A PGPR2 genomic region (1587 bp; Genbank accession number ASQO01000097.1, region 3154 : 1568) was identified as DEAD/DEAH-box helicase and shared 81% identity with a helicase from* Pseudomonas stutzeri* A1501 in BlastP analysis.

### 3.11. Receptor for Ferrienterochelin

A unique region (1271 bp, Genbank accession number ASQO01000059.1, regions 111 : 1382) coding for ferrienterochelin receptor—FepA was identified on the PGPR2 genome. FepA is an 81,000-dalton protein in the* E. coli* outer membrane that functions in the initial step of iron uptake by binding ferrienterochelin [39]. Receptors for siderophores such as ferrienterochelin are necessary for survival in the rhizosphere by competition. The ability to uptake self siderophores and siderophores that are secreted by other competing bacteria provides an advantage for PGPR2 in the rhizosphere. This may subsequently lead to depletion of iron, leading to suppression of pathogen growth in the immediate vicinity of the plant.

### 3.12. Antifungal Proteases

Nearly 60 loci on the PGPR2 genome were annotated as different types of proteases. Their molecular weight ranged from 4000 to 100,000 Da. Among 60 sequences only two proteases showed molecular weight in the range 30 to 34 kDa. Earlier, we have identified and characterized a proteolytic enzyme with a molecular weight of ~33 kDa. This protein exhibited efficient antifungal activity. Peptide mass fingerprinting results showed that the mass value matched with the amino acid sequence of protease HtpX of* Pseudomonas aeruginosa* strain PA7. The two proteases of interest on the PGPR2 genome (molecular weights of 31592.92 and 34389.2 Da) showed considerable sequence homology with a probable protease HtpX homolog and a putative cysteine protease, respectively. A genomic region (876 bp, ASQO01000001.1, regions 2206178_2205303) was found to encode a homolog of the protease HtpX of* Pseudomonas aeruginosa* strain PGPR2.

### 3.13. Antifungal Secondary Metabolites from PGPR2

**Figure 3:**
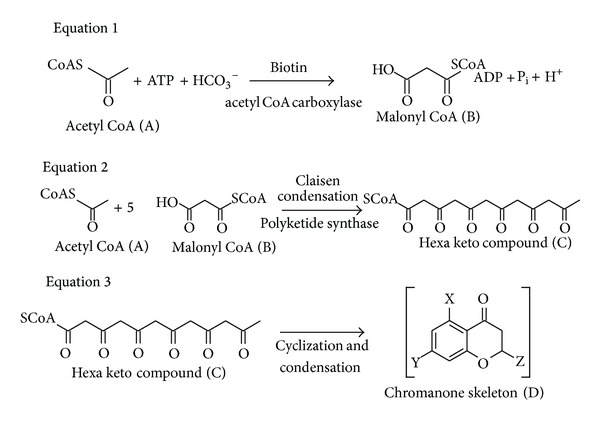
Pathway proposed for production of the antifungal metabolite, 3, 4-dihydroxy-N-methyl-4-(4-oxochroman-2-yl) butanamide by PGPR2. Equation 1: Acetyl CoA carboxylase catalyzed the formation of malonyl CoA from acetyl CoA; Equation 2: Polyketide synthase catalyzed the formation of hexa keto compound by condensation of a molecule of acetyl CoA with five molecules of malonyl CoA; Equation 3: The hexa keto compound undergoes cyclization and condensation reactions to form different chromanone intermediates.

Genes belonging to metabolic pathways involved in antifungal activity were identified from the genome of PGPR2. These metabolites include phenazine, pyocyanin, paerucumarin, pyoverdine, pyochelin, hydrogen cyanide, AHLs, rhamnolipids, phenylacetic acid, and macrolide antibiotics. In addition, we have demonstrated earlier that 3, 4-dihydroxy-N-methyl-4-(4-oxochroman-2-yl) butanamide, a secondary metabolite synthesized by PGPR2, showed antagonism against* Macrophomina phaseolina *[7]. Based on the survey of the PGPR2 genome putative biosynthetic pathway was identified. One molecule of acetyl CoA can condense with five molecules of malonyl CoA, which in turn can be obtained by the action of acetyl CoA carboxylase with biotin as the cofactor (Equation 1), to give a hexa keto compound (Equation 2), which can then be cyclised to give different chromone intermediates (Equation 3) which is represented in Figure 3. The basic chromanone skeleton of 3, 4-dihydroxy-N-methyl-4-(4-oxochroman-2-yl) butanamide could have been generated by a similar reaction. However, the mode of dehydroxylation of the aromatic ring of the constructed chromanone unit and the route by which the side chain moiety is obtained and gets itself attached to the 2-position of chromanone ring are still unclear.

### 3.14. Other Features of* P. aeruginosa *PGPR2 Genome Relevant to Its Plant Growth Promoting Ability

#### 3.14.1. ACC Deaminase Synthesis

In addition to PGPR2, the genomes of M18, LesB58, PAO1, PA7, DK2, and UCBPP-PA14 contained a putative 1-aminocyclopropane-1-carboxylate (ACC) deaminase which showed 100% homology in BlastP analysis (899 bp, Genbank accession number ASQO01000001.1, regions 1920268 : 1921167).* P. aeruginosa* strains containing 1-aminocyclopropane-1-carboxylate deaminase (ACCD) are capable of growing on ACC as a nitrogen source. These organisms when colonized on the surface of plant roots break down the ACC exuded from the plant to ammonia and *α*-ketobutyrate. This in turn is advantageous for the plant as these organisms sequester ACC thereby lowering the level of the stress hormone ethylene in plants [40].

#### 3.14.2. Siderophores Synthesis

The PGPR2 genome encoded a number of genes involved in iron acquisition such as the siderophore pyoverdine, one of the major siderophores found in all fluorescent pseudomonads. In addition, all the genomes contain the gene coding for TonB. This protein spans the periplasm and is anchored to the cytoplasmic membrane interacting with receptors in the outer membrane to facilitate the uptake of iron-siderophore complexes. Thus, the production of active siderophores could contribute to potent biocontrol property against soil-borne pathogens. Earlier reports have suggested that* P. fluorescens* Pf-5 and* P*.* aeruginosa* M18 contained genes (*pch*) encoding a secondary siderophore pyochelin, which has antifungal activity [41]. The pyochelin biosynthetic gene cluster (*pchCEFGR*) was identified using BlastP analysis in all chosen* P. aeruginosa *strains (Table 2). Interestingly, the strain PGPR2 contained the complete pyoverdine (Pvd) biosynthetic gene cluster (*pvdJAEGXSLHIONMQPY*), whereas one or more of these genes were missing in other genomes (Table 2). The ability of bacteria to produce multiple siderophores benefits them, as they may function in different environments, making them more competitive against other organisms in the same niche.

#### 3.14.3. Indole Acetic Acid Production

Pseudomonads, fluorescent strains in particular, have been known to synthesize the phytohormone indole acetic acid (IAA). IAA is the most common and best characterized phytohormone. It has been estimated that 80% of bacteria isolated from the rhizosphere produce IAA [42]. However, the amount of IAA produced may vary significantly among strains. Based on the concentration, IAA can either stimulate or inhibit plant growth. The production of indole-3-acetic acid by rhizobacteria has been associated with plant growth promotion, especially root initiation and elongation. The role of bacterial IAA in suppression of charcoal rot disease of chickpea has been reported [43]. A genomic region containing an 837 bp CDS was found to have homologs in all chosen* P. aeruginosa *strains with 100% identity using BlastP analysis. This region was annotated as indole-3-glycerol phosphate synthase (ASQO01000001, regions 682275-683111), an enzyme responsible for the synthesis of IAA.

#### 3.14.4. Trehalose Synthesis

Trehalose is a nonreducing disaccharide present in a wide variety of organisms. Certain species of plant, fungi, bacteria, and invertebrates are known as anhydrobionts due to the production of trehalose. This enables them to revive themselves in the presence of water in a few hours after being completely dehydrated for months or years [44]. Rodríguez-Salazar et al. [45] reported the effects of genetically engineered* Azospirillum brasilense* for trehalose biosynthesis conferred drought tolerance and also significantly increased leaf and root biomass of maize plant. The PGPR2 genome encoded a maltooligosyl trehalose synthase gene* treY *(accession number ASQO01000001, regions 3205742-3206398), Maltooligosyl trehalose hydrolase gene* treZ*, (accession number ASQO01000001, regions 3199525-3201276), and trehalose synthase gene* treS* (accession number ASQO01000001, regions 3219761-3216459), required for biosynthesis and accumulation of trehalose, a metabolite that is of key importance in conferring tolerance to low water environments. The homologs of these genes were found in all* P. aeruginosa *strains with 100% identity in BlastP analysis.

#### 3.14.5. Acetoin and Butanediol Production

Certain bacterial species ferment pyruvate to acetoin and 2,3-butanediol through 2-acetolactate. Pyruvate is converted into either lactate by the enzyme L-lactate dehydrogenase or D-lactate dehydrogenase or to 2-acetolactate by the enzyme *α*-acetolactate synthase (*α*-ALS) [46]. Genes coding for the enzymes involved in acetoin and 2,3-butanediol synthesis were detected using BlastP in the genome of PGPR2 (182 bp, accession number ASQO01000001, region 5377021-5376839). The conversion of pyruvate to lactate requires NADH and when the levels of NADH are limited, a majority of the pyruvate is converted to 2-acetolactate. Since 2-acetolactate is an unstable intermediate, it undergoes spontaneous decarboxylation in the presence of oxygen, producing diacetyl. Once diacetyl is formed, it can be converted to acetoin by the activity of the enzyme diacetyl reductase (755 bp, accession number ASQO01000016.1, regions 144-899) (also known as acetoin dehydrogenase). Under anaerobic conditions 2-acetolactate is converted directly to acetoin by *α*-acetolactate decarboxylase (*α*-ALD).* P. aeruginosa* strains depend on acetoin dehydrogenase for synthesis of acetoin rather than using *α*-acetolactate decarboxylase. The production of acetoin and 2,3-butanediol by plant growth promoting bacteria was reported to increase systemic disease resistance [47] and drought tolerance [48].

#### 3.14.6. Hydrogen Cyanide Production

HCN, a secondary metabolite produced in* Pseudomonas* spp., is catalyzed by the membrane-bound enzyme HCN synthase, which forms HCN and CO_2_ from glycine [49]. The* hcnABC *genes encoding HCN synthase (2961 bp, accession number ASQO01000001.1, regions 3175436-3172475) were identified on a genomic fragment of* P. aeruginosa* PGPR2. A blastp search using the region as query identified homologs in all other strains of* P. aeruginosa*. Lanteigne et al. [50] isolated HCN producing* Pseudomonas* sp. LBUM300 and observed effective biological control activity against bacterial canker of tomato. HCN production in* P. aeruginosa *PGPR2 was verified as described earlier by Bakker and Schippers [51].

#### 3.14.7. Chitinase Production n

Chitinases produced by* Pseudomonas spp*. can hydrolyze the *β*-1,4-linkages in chitin, an abundant N-acetyl-*β*-D-glucosamine polysaccharide which is an integral structural component of fungal cell walls. Thus, bacterial chitinases contribute to the biological control of fungal phytopathogens [52].* P. aeruginosa* PGPR2 encodes a chitinase (1451 bp, ASQO01000001.1, region 2978066-2979517). Similarly, using BlastP, all the strains of* P. aeruginosa *were found to encode at least one chitinase.

#### 3.14.8. Acyl Homoserine Lactone Synthesis

*P. aeruginosa* PGPR2 produce N-acyl-homoserine lactone (AHL), a well-known quorum sensing signalling molecule. Earlier studies have reported the involvement of these molecules in plant growth promotion and protection against salt stress [53]. As quorum sensing is known to modulate population dependent genes including virulence factors, it is necessary to study the means of interference with quorum sensing systems. Interruption of quorum sensing, known as quorum quenching, is usually accomplished by production of lactonases or acylases. Lactonases inactivate AHLs by hydrolyzing the ester bond of the lactone ring while acylases cleave the amide bond that connects the lactone moiety and acyl side-chain. The products of the enzymatic breakdown do not function as active signal molecules. As described earlier, the* P. aeruginosa* PGPR2 genome encodes* pvdQ *(2289 bp, ASQO01000001.1, regions 2872266-2872652) involved in the production of acyl-homoserine lactone acylase. Earlier studies have shown that the PvdQ prefers long c-length AHL molecules as a substrate [54]. The overexpression of PvdQ in* P. aeruginosa* strains is shown to inhibit the accumulation of signal molecules (3-oxo-C12-HSL and 2-heptyl-3-hydroxy-4(1H)-quinolone) and thereby decreases expression of several virulence factors such as elastase and pyocyanin that are important for* P. aeruginosa* pathogenicity in animals [55].

## 4. Conclusion

In this study, the genome of the* P. aeruginosa *strain PGPR2 was sequenced and analyzed. The draft genome PGPR2 possesses an array of genes encoding unique functions corresponding to its plant growth promotion and biocontrol abilities, besides sharing a common core genome to other sequenced strains of this species. Interestingly, the strain PGPR2 genome has acquired several genes needed for the rhizopheric environments from other organisms, which are not found in other medically relevant* P. aeruginosa *strains.

## Figures and Tables

**Table 1:** Features of the *P. aeruginosa* PGPR2 genome in comparison with *P. aeruginosa* strains from other ecological niches.

Niche	PGPR2	LESB58	PA7	PAO1	UCBPP-PA14	DK2	M18
	Mung bean rhizosphere #	Cystic fibrosis patient #	Cystic fibrosis patient #	Human burn wound #	Human burn wound #	Cystic fibrosis patient #	Sweet melon rhizosphere #
Total number of genes	**6883**	6061	6369	5697	5977	5959	5770
PEGs	**6803**	5925	6286	5566	5892	5884	5684
5S rRNA	**4**	4	4	4	4	4	4
16S rRNA	**4**	4	4	4	4	4	4
23S rRNA	**4**	4	4	4	4	4	4
tRNA genes	**68**	67	63	63	59	64	61
misc_RNA	**0**	21	0	4	0	0	5
Predicted genes	**5314**	4172	3638	3248	3583	3972	4116
Hypothetical genes	**1489**	1753	2648	2318	2309	1912	1568
CRISPR repeats	**3**	1	2	1	2	3	3

# Count.

**Table 2:** Genes associated with pyoverdine synthesis in *P. aeruginosa* strains.

Gene	Function	PGPR2	M18	DK2	LESB58	UCBPP-PA14	PAO1	PA7
*pvdj *	Pyoverdine sidechain nonribosomal peptide synthetase	+	+	+	+	+	+	+
*pvdX *	Hypothetical protein	+	+	+	+	+	+	+
*pvdS *	Sigma factor controlling pyoverdine biosynthesis	+	+	+	+	+	+	+
*pvdL *	Pyoverdine chromophore precursor synthetase	+	+	+	+	+	+	+
*pvdH *	L-2,4-diaminobutyrate: 2-oxoglutarate aminotransferase	+	+	+	+	+	+	+
*pvdI *	Pyoverdine sidechain nonribosomal peptide synthetase	+	+	+	+	+	+	+
*pvdO *	Pyoverdine responsive serine/threonine kinase	+	+	+	+	+	+	+
*pvdN *	Putative aminotransferase, class V	+	+	+	+	+	+	+
*pvdM *	Putative dipeptidase	+	+	+	+	+	+	+
*pvdQ *	Acyl-homoserine lactone acylase	+	+	+	+	+	+	+
*pvdY *	Hypothetical protein	+	+	+	−	−	−	+
*pvdG *	Thioesterase involved in nonribosomal peptide synthesis	+	+	+	+	+	+	−
*pvdE *	Pyoverdine ABC export system: ATPase and permease components	+	+	+	−	−	−	+
*FpvB *	Outer membrane ferripyoverdine receptor FpvB, for Type I pyoverdine	+	+	−	+	+	+	−
*pvdP *	Pyoverdine biosynthesis related protein	+	+	+	+	−	−	+
*pvdA *	L-ornithine 5-monooxygenase	+	−	+	+	+	+	+
